# Measurement of Connectedness with Nature: Evidence of Validity and Reliability for Use in Colombian Urban and Rural Sustainability Contexts

**DOI:** 10.3390/ijerph22081185

**Published:** 2025-07-29

**Authors:** Willian Sierra-Barón, Andrés Gómez-Acosta, María Delfina Luna-Krauletz, Sergio Falla-Tapias, Erika Judith López-Santamaria

**Affiliations:** 1 Sintropia Research Group, Surcolombiana University, Neiva 410003, Colombia; erika.lopez@usco.edu.co; 2 Department of Psychology, University of Pamplona, Pamplona 543050, Colombia; cesar.gomez@unipamplona.edu.co; 3 Instituto de Estudios Ambientales, Universidad de Sierra Juárez, Oaxaca 68725, Mexico; mkrauletz@unsij.edu.mx; 4 Program of Veterinary Medicine and Zootechnics, Faculty of Veterinary Medicine and Related Sciences, Corporación Universitaria del Huila (Corhuila), Neiva 410003, Colombia; sergio.falla@corhuila.edu.co

**Keywords:** connectedness with nature, sustainability, urban–rural contexts, psychometrics, pro-environmental behavior, well-being

## Abstract

The growing disconnection between humans and nature—particularly in urban environments—has been associated with declining well-being and lower engagement in pro-environmental behavior. Although the Connectedness with Nature Scale (CNS) has been widely used internationally to measure this relationship, there is a lack of evidence on its validity and reliability in Latin American contexts, especially in urban and rural settings. This study aims to address this gap by examining the psychometric properties of the CNS in a sample of 956 Colombian participants. Using exploratory and confirmatory factor analyses, we tested two versions of the scale (14-item and 12-item models), both showing good fit and high internal consistency (α > 0.90). Convergent validity was confirmed through strong correlations with the Environmental Identity and Pro-environmental Behavior Scales. These findings support the CNS as a valid and reliable tool to assess the human–nature connection in Colombia and highlight its potential for informing urban sustainability initiatives, environmental education, and public policy in diverse sociocultural contexts.

## 1. Introduction

The link between humans and nature has been fundamental throughout the evolutionary process on Earth, given the inherent need to depend on it for subsistence [1]; over time, this link has been modified in various ways according to cultural and historical contexts [2,3].

Currently, there is debate around the concept that dependence between humans and nature may have led to increased environmental problems [4,5,6], such as biodiversity loss, land-use changes, and environmental degradation [7,8,9,10] and, although this needs to be addressed in greater depth, what is clear is the need to understand the implicit factors underlying the ways in which people live and experience (or not) connectivity with nature.

Connection to nature is a psychological construct that describes the degree to which individuals feel emotionally, cognitively, and behaviorally linked to the natural environment [3,11,12,13,14,15,16,17]. This connection is considered a key factor in promoting pro-environmental attitudes and behaviors, as well as people’s well-being [18,19,20].

In recent years, this construct has become especially relevant in the context of urban sustainability, given the growing disconnect between people and natural environments due to urbanization, digital lifestyles, and the limited presence of green spaces in cities. This disconnection not only threatens ecological awareness but also undermines individual and collective well-being, which reinforces the need for reliable tools to assess this relationship in diverse socio-environmental contexts.

Connectivity with nature also plays a crucial role in the realm of mental health, as those with a stronger connection to nature experience lower levels of stress and anxiety, especially when they frequently visit public green spaces, highlighting the psychological benefits of contact with nature [21], since it influences problem-focused coping strategies, which indirectly reduces stress and depression [22] or, in the case of bereavement, where the feeling of a stronger connection with nature helps to mitigate the negative effects of losses suffered by individuals [23].

Prolonged exposure to nature has been shown to contribute to increased brain activity with positive effects on cognitive function and a reduction in stress-related physiological markers such as blood pressure and cortisol levels, further reinforcing its role in mental health [24]. On the other hand, during the COVID-19 pandemic, connection with nature was found to significantly predict lower levels of depression and stress, highlighting its importance in promoting mental health in times of crisis [25].

These studies show that the effects are moderate to high, indicating a positive correlation with various areas of well-being, including emotional, psychological, and social aspects [26]. In addition, spirituality has been identified as mediating the relationship between connection to nature and well-being, suggesting that spiritual experiences in nature may improve health outcomes [27]. The implications of nature connection extend to planetary health as well, as it fosters prosocial attitudes and pro-environmental actions, which are essential to address the aforementioned global challenges [28].

However, recently it has been observed that people’s connection with nature is increasingly decreasing [29], a situation that could be related to increasing urbanization that reduces everyday experiences with nature [30,31], coupled with limited access to green spaces [32,33] and increased interaction with virtual reality mediated by devices such as cell phones and computers [30,34,35], leading people to spend more time indoors and reducing direct contact with the natural environment [36,37].

In the context of accelerated urbanization, the separation between people and nature becomes more evident, requiring the development of valid and reliable tools to measure this relationship. The Nature Connection Scale is one of the tools that has been widely used in Anglo-Saxon contexts to assess the emotional and experiential link between humans and nature. However, its applicability in diverse urban settings is limited due to its original design in rural and culturally homogeneous settings [38,39].

In particular, previous studies have identified conceptual ambiguities in the Connectedness with Nature Scale (CNS), and difficulties in capturing variability across sociocultural groups, especially between urban and rural populations [40,41]. This raises the need for adaptation and validation in different national and ecological contexts.

Some research has shown that a greater connection with nature is associated with higher levels of environmental concern, a willingness to conserve, and more active participation in sustainable practices [6,42]. Despite this, available tools to assess connectivity with nature often lack cultural sensitivity and adaptability to urban contexts, as demonstrated by mixed results of CNS brief invariance in different European cities, which highlight its sensitivity to linguistic and cultural contexts [40]. Furthermore, urbanization affects human–nature connectivity by weakening it in urban and urbanized areas compared to rural areas, although it remains positively correlated with perceived values of nature at different levels of urbanization [41].

This emphasizes the need to develop measurement tools that are not only reliable but also culturally and contextually adaptable to effectively understand the nuances of human–nature relationships in urban settings. Additionally, factors such as place of residence, age, and cultural background significantly influence nature connectivity, suggesting that tools must be adapted to account for these variables to improve their validity and applicability in diverse urban settings [6,43].

In Colombia, despite increasing academic and policy interest in environmental education and sustainability, no psychometric validation of the CNS has yet been carried out. This represents an empirical and methodological gap, particularly considering the country’s ecological diversity and territorial inequalities.

Therefore, the present study aims to validate the Connectedness with Nature Scale (CNS) in the Colombian population across both urban and rural settings. This will allow for the use of a reliable and culturally sensitive instrument to evaluate this construct in Latin American contexts, and to support the design of environmental education strategies and sustainability policies based on solid evidence.

## 2. Materials and Methods

### 2.1. Type of Study

An instrumental–psychometric study [44] was carried out, which involves the analysis of psychometric properties [45], through sequential phases that included an exploratory factor analysis (EFA) to examine the underlying structure, followed by a confirmatory factor analysis (CFA) to test the theoretical model, whose purpose was to determine the evidence of reliability and validity of the “Nature Connection Scale” by Mayer and Frantz [12] in the Colombian population. The study was designed in accordance with international standards for the validation of scales in applied psychology.

### 2.2. Participants

A sample of 957 persons (58.8% women) selected by intentionality and convenience participated in this research; however, even though the sampling is broad, an application with probabilistic criteria can be carried out in the future to ensure greater generalization potential. The mean age of the participants was 32.75 years (SD 12.32 years). A total of 54.6% (523) identified themselves as urban dwellers, while 434 people (33.92%) reported belonging to a rural context (Table 1).

### 2.3. Instruments

Ad Hoc Questionnaire for sociodemographic variables (information on sex, age and origin).

Connectivity with Nature Scale [12]. This scale comprises 14 items with five response options (1 = strongly disagree and 5 = strongly agree). It measures how people include nature as part of their cognitive representation. Olivos-Jara et al. [38] translated and adapted the scale to Spanish, showing a Cronbach’s α of 0.70 for the general population and 0.75 for the university population (Appendix A). The purpose of this research is to evaluate whether this scale translated by Olivos-Jara et al. [38] has adequate psychometric properties to measure connectivity with nature with the Colombian population in a suitable way. 

In addition, the following instruments were used to identify convergent validity:

Environmental Identity Scale [46]—This scale contains 24 items with 5 response options from 1 to 5 (1 = strongly disagree and 5 = strongly agree). Olivos and Aragonés [47] adapted the scale to Spanish, showing a Cronbach’s α of 0.89, proposing a factor structure underlying the scale, composed of four dimensions: “Environmental Identity” itself, which is related to a self-reflection that refers to a sense of belonging to the natural world, in the same sense of connection with nature; “Enjoying Nature”, which refers to contact with nature, mainly in outdoor activities, and the pleasure or benefit that this brings at an individual level; “Appreciation of Nature”, which expresses the valuation of the natural environment due to the attribution of unique and complex qualities such as beauty, spirituality or personality; and “Environmentalism”, a form or style of behavior and commitment to the environment that appeals to a moral code, an ideological commitment or an identification with environmentalists.

Pro-environmental Behavior Scale [48]—This scale is made up of 11 items with five response options from 1 to 5 (1 = strongly disagree and 5 = strongly agree). It consists of three underlying dimensions of the scale that could reflect three different types of behavior [49]: “Ecologist” (items = 8, 9, 10, and 11), which includes environmental volunteering behaviors and ecological consumption options; “Urban” (1, 2, and 3), which includes appropriate waste disposal and care of parks and gardens; and “Camper” (4, 5, 6, and 7), which incorporates behaviors to protect the environment while camping and during leisure activities. The instrument presents acceptable internal consistency indicators (α > 0.50) for each dimension.

### 2.4. Procedure

The scale of connectedness with nature, together with the other instruments, was administered in person, in pen and paper format, to participants who voluntarily agreed to take part in the study after being informed of the research objectives and signing the corresponding consent form. The ethical provisions for research with humans indicated both by the American Psychological Association in its stated codes and ethical standards (2017) (Sections 4, 8 and 9), as well as by Resolution 8430 of 1993 and Law 1090 of 2006 in Colombia, referring to psychological research and research with human beings, were included. The study did not pose any risk to the participants, who did not receive any financial compensation. The specific results were communicated individually to those who explicitly requested them [50].

### 2.5. Data Analysis

Before conducting the factor analyses, the required statistical assumptions were assessed. Sampling adequacy was confirmed using the Kaiser–Meyer–Olkin (KMO) index (0.959) and Bartlett’s test of sphericity (χ^2^ = 8677.24; df = 91; *p* < 0.001), indicating the data were suitable for factor analysis.

The total sample (*n* = 956) was randomly divided into two equally sized subsamples (*n* = 478 each). The first subsample was used to conduct an exploratory factor analysis (EFA) using the principal axis factoring method, with direct Oblimin rotation, based on the assumption of factor correlation. This method was selected instead of principal component analysis (PCA), as it better identifies shared variance among items—a critical criterion for the psychometric validation of latent constructs.

The second subsample was used for a confirmatory factor analysis (CFA) using structural equation modeling, in order to test the theoretical model. The analysis was conducted using JAMOVI version 2.3, based on the R Project statistical (version 4.5.1) environment, which enables the robust estimation of measurement models via maximum likelihood.

Model fit was evaluated using the following indices: CFI and TLI ≥ 0.90, RMSEA ≤ 0.08, SRMR ≤ 0.05, and χ^2^/df ≤ 3.00, in accordance with established psychometric standards. Two versions of the scale were compared: the original 14-item version and a shortened 12-item version, both assessing a one-dimensional structure.

Missing data accounted for less than 2% of the total dataset and were handled through pairwise deletion. No systematic patterns of missingness were detected. Measurement invariance of the scale was tested through multigroup CFA by sex (male/female) and place of residence (urban/rural). Configural, metric, and scalar models were applied, and model stability was evaluated following the guidelines of Chen (2007) [51], namely ΔCFI < 0.01 and ΔRMSEA < 0.015, between nested models.

Items 4 and 14 of the original CNS versions showed low factor loadings in the CFA; however, they were retained in the initial model for comparative purposes. After excluding them, the 12-item model showed improved fit indices and is thus proposed as the most robust version for use in the Colombian context, without compromising the scale’s construct validity or internal consistency.

## 3. Results

Initially, the analysis of initial psychometric properties was carried out, and subsequently, the factorial invariance analysis was performed with the structural equation technique.

For the first moment, when performing the principal components analysis, adequate factorial weights were identified (above 0.70), except for items 4 and 14, which showed minimal factor loadings (not statistically significant) and low correlations with the rest of the items of the instrument. In addition, these items present more general or abstract formulations with respect to the content of the construct, which could have affected their coherence with the factor structure in this specific sample.

An extraction above 0.50 and a test item correlation above 0.40 were also evidenced, corresponding to adequate indicators. On the other hand, it was determined that, in total, the test presents 61.28% of the accumulated variance of the construct measured. The details of this analysis are recorded in Table 2.

**Figure 1:**
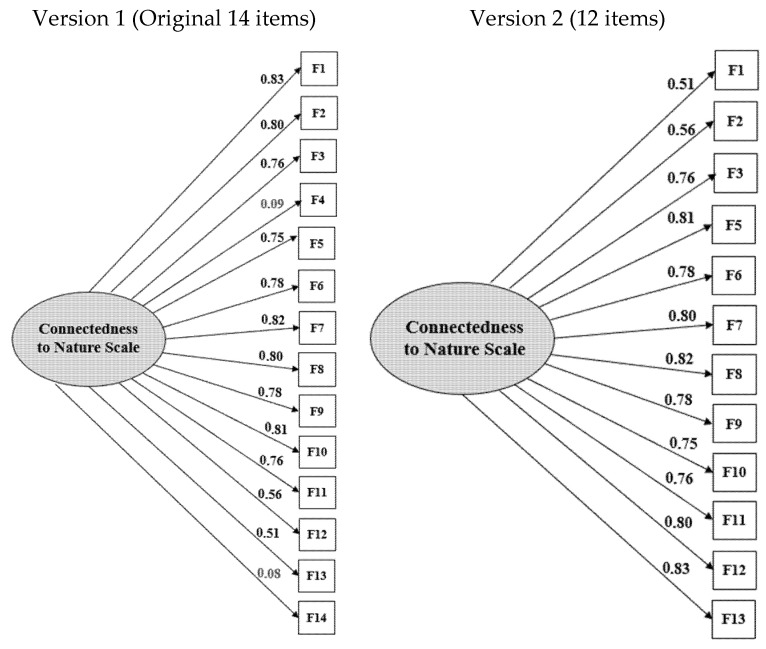
Equation model (Original 14-item and 12-item versions).

For the confirmatory analysis (Figure 1), two alternatives are presented (with fourteen items and eliminating the two previously mentioned items). Models with adequate goodness-of-fit indicators were obtained for RSMEA and SRMR, as well as for the CFI and TLI indices, these being slightly higher for the 12-item proposal; as for X^2^/gl, the coefficient obtained also fits better in the 12-item model (Table 3).

A multigroup factor analysis was carried out to evaluate the invariance of the Connection with Nature Scale with the 12-item version, as a function of sex and origin of the participants. As shown in Table 4, for the sex criterion, all assumptions are met in the unrestricted and restricted models (metric, scalar, and structural invariance), indicating total invariance. In the case of origin, although the configural model complies with the established assumptions, the results show partial invariance in the metric and scalar models, which suggests that the factor structure of the scale is maintained in general terms, although with certain differences according to the context of origin (urban or rural).

Similarly, the 12-item version of the scale correlates with high, positive and statistically significant indicators with the Environmental Identity Scale (Spearman’s Rho = 0.82) and the Pro-environmental Behavior Scale (Spearman’s Rho = 0.62), demonstrating convergent validity.

Finally, the estimation of the internal consistency of the total test Cronbach’s α (0.90) and McDonald’s ω (0.92) in the 14-item version, and also Cronbach’s α (=0.93) and McDonald’s ω (0.94) in the 12-item version, reported optimal levels, so it can be concluded that the measures obtained with the test are highly reliable.

## 4. Discussion

The study of the evidence of validity and reliability of the scales that measure the connection with nature is fundamental due to the limited number of works that exhaustively demonstrate these psychometric properties, and the need to have adequate instruments for decision making. The Connection with Nature Scale (CNS) developed by Mayer and Frantz [12] is the most frequently used tool in this field, and even though it has been validated in various contexts and countries such as Spain, Ecuador, Mexico, and Italy, among other countries, where its effectiveness has been proven. No previous work has been documented in Colombia.

This absence represents an important empirical gap in the Colombian context where there is a growing interest in strengthening environmental education, promoting Pro-environmental Behaviors and informing public policies that address sustainability challenges in urban and rural contexts. In Colombia, the link with nature is mediated by geographic and cultural diversity, as well as historical and structural differences between rural and urban environments.

For example, the results of this study are consistent with the findings reported previously; it shows a higher total cumulative variance of the construct (61.28%), in comparison with the original version of Mayer and Franz (32%) and the translation of Olivos-Jara et al. to Spanish. In addition, when compared with the version of Olivos-Jara et al. [38], higher indicators of internal consistency (α = 0.75) are obtained, although in our case, confirmatory analysis statistics were applied with more precise goodness-of-fit indicators, not used in the Spanish version. Likewise, goodness-of-fit indicators are slightly higher than those identified in the French [52] and Italian [39] versions, and similar to those tested in seven European cities [40].

When compared with the French version [52], difficulties of fit were also found with items 4 and 14 (although in the latter, this situation was also identified with item 12); however, it also reports a unifactorial structure, adequate internal consistency (α = 0.85), although higher in the Colombian version) and convergent validity with the Environmental Identity Scale. On the other hand, the Italian version [39] retains the original 14 items and also has a high internal consistency (α = 0.89). It is important to highlight that in this study, an analysis of invariance by sex and by origin (urban/rural) was incorporated, an aspect that was not evident in any other of the studies reviewed, which made it possible to evaluate the stability of the test as a function of these dichotomous variables.

Two structures were obtained in the analysis, the first version with 14 items (original) and one with 12 items, eliminating the two items that do not contribute to the variance of the construct according to the exploratory analysis; both structures present an optimal fit to the theoretical model proposed in the original study. This is reflected in the χ^2^/df, CFI, and TLI indicators, which would allude to the correspondence of the data with the evaluated hypothetical structure, as well as with the RMSEA and SRMR indices with low magnitudes, which represents an adequate concordance between the expected covariances and those estimated by the model [53]. Similarly, for both versions, adequate Cronbach’s α values were obtained (0.90 for the 14-item version and 0.93 for the 12-item version), indicating high reliability, even higher than the reports for the Italian (α = 0.84) and Spanish (α = 0.78) versions. In this respect, it can be inferred that the items show a high internal correlation without the presence of redundancy, which is an indicator of the stability of the measurement [53]; however, it will be necessary to compare it with at least one test–retest application, which will make it possible to confirm that this stability is maintained over time.

Concurrent validity is a crucial aspect for evaluating the efficacy of scales that measure connection with nature, as it guarantees that the scale correlates adequately with other established measures of the same construct. In the present study, the concurrent validity analysis was carried out with the Environmental Identity Scales (Spearman’s Rho = 0.82) and the Pro-environmental Behavior Scale (Spearman’s Rho = 0.62), showing a correlation with high, positive and statistically significant indicators, ensuring convergent validity. Such validations are essential, as they confirm that these scales not only measure the intended construct but also align with related psychological variables and it is vital to ensure that scales measuring connection with nature are robust, reliable and applicable in diverse populations and contexts, thus supporting their use in both practical and research applications aimed at fostering sustainability and environmental responsibility [20].

On the other hand, various factors such as gender, age, and place of residence significantly influence the connection with nature [54], as shown by studies carried out in Spain and Ecuador, where urban and rural contexts affected the perception of nature [43]. In this study, a multigroup factor analysis was carried out to test invariance, according to the sex and origin of the participants, in which it was possible to show in the unrestricted and restricted models (configural, metric, scalar, and strict) that it fulfills all the assumptions for the sex item, but only fully complies with the invariance parameters in the case of the unrestricted model, and partially for the metric and scalar restriction models in the case of origin (rural/urban). This suggests that the CNS can identify “real” differences in the connection with nature between men and women free of bias due to the structure of the instrument [55]; on the other hand, although the ΔX2 is not as expected in the comparison by origin (rural/urban of the sample), it could be accepted that the instrument maintains the factor structure according to this sociodemographic criterion if the obtained ΔCFI and ΔRMSEA indicators are taken into account [51,56].

In relation to partial invariance by origin, it could reflect differences in the way people in urban and rural contexts experience and conceptualize their connection with nature. For example, those living in rural areas may have more frequent and direct interactions with natural environments, while in urban contexts nature may be perceived in a more symbolic way. These differences may influence the way in which scale items are interpreted and valued.

The results of this psychometric analysis should be interpreted with caution in light of the following limitations: first, the sampling is large, but in the future an application with probabilistic criteria can be carried out to ensure a greater potential for generalization; on the other hand, biases in the responses related to social desirability cannot be ruled out, given that some participants may estimate this psychological characteristic with higher scores in the presence of the evaluator [57]. To minimize this limitation, it is proposed that future studies incorporate validated measures to control this type of bias, such as subscales of the Eysenck Personality Inventory [58], items from the Beck Depression Inventory [59], or the use of the Marlone–Crowne Social Desirability Scale [60] that include items sensitive to this type of response (although it is clarified that there are no adaptations of them to the Colombian context specifically). Another alternative is to extend the original Likert scale with a wider range of values than originally proposed [61].

In addition, it is recommended that future studies include test–retest validation procedures in order to confirm that the scale consistently measures connection with nature over time. From a theoretical perspective, the connection with nature has been understood as an emotional, cognitive, and behavioral experience [12], which can be deeply influenced by cultural and contextual factors. In the Colombian case, the marked division between urban and rural areas not only reflects geo-graphic differences but also different ways of living and relating to nature. In this sense, the results obtained not only support the validity and reliability of the Colombian version of the CNS, allowing the possibility of its application in future research and in practical contexts; for example, this scale can be used by researchers to identify possible differences in the connection with nature according to the different populations that inhabit regions as dissimilar as the coastal region, the Andean region or the Amazon jungle, to cite a few ecosystems.

Additionally, with this instrument, profiles such as teachers and professionals in environmental areas can collect useful information to characterize the population and develop environmental education programs, sustainability interventions, public policy formulation, and impact evaluations. In particular, the CNS could play an important role in marginalized communities or territories affected by armed conflict, by facilitating strategies for reconnecting with the natural environment as a symbolic and emotional resource for rebuilding the social fabric. It can also support sustainable urban planning processes, promoting more resilient and equitable environments. The CSN, with psychometric properties tailored to the Colombian context, becomes an essential baseline tool, enabling diagnoses and evaluations of the impact of interventions aimed at strengthening environmental sustainability and ecological education. It can also contribute to the design of strategies for reconnecting with nature through artistic initiatives or narratives that incorporate cultural values.

## 5. Conclusions

The evidence provided allows us to conclude that the translation of the CNS works adequately for the measurement of connection with nature in the Colombian context, as it demonstrates appropriate psychometric properties of content validity, construct validity and internal consistency, as well as presenting a tendency to maintain its factor structure invariant according to the sex of the population. These findings highlight the importance of considering cultural aspects and methodological rigor when developing, as well as validating, instruments to measure the connection with nature, ensuring that they are reliable and culturally sensitive.

## Figures and Tables

**Table 1:** Sociodemographic characteristics of the participants (*n* = 957).

Age	M (SD)
Woman (n = 563; 58.8%)	31.83 (0.49)
Man (n = 394; 41.2%)	34.03 (0.65)
Total n = 957	32.75 (12.32)
**Provenance**	**F/%**
Urban	523 (54.6%)
Rural	434 (45.4%)
**Engages in activities involving contact with nature**	**F/%**
yes	743 (77.6%)
No	214 (22.4%)
**Frequency with which you carry out activities in nature**	**F/%**
Occasionally	161 (16.8%)
Sometimes	304 (31.8%)
Often	214 (22.4%)
Always	64 (6.7%)
Not applicable	214 (22.4%)

**Table 2:** Characteristics of the items and subscales.

Item	F1	Sig.	Extraction	Item-Test Correlation
EIA1	0.8396	<0.001	0.705	0.777
EIA2	0.8202	<0.001	0.677	0.767
EIA3	0.7849	<0.001	0.627	0.704
EIA4	**0.0955**	**0.008**	0.663	**0.052**
EIA5	0.7794	<0.001	0.617	0.724
EIA6	0.8034	<0.001	0.651	0.748
EIA7	0.8373	<0.001	0.702	0.769
EIA8	0.8184	<0.001	0.676	0.741
EIA9	0.8066	<0.001	0.651	0.740
EIA10	0.8248	<0.001	0.683	0.755
EIA11	0.7889	<0.001	0.623	0.722
EIA12	0.5996	<0.001	0.421	0.549
EIA13	0.5499	<0.001	0.334	0.464
EIA14	**0.0912**	**0.015**	0.552	**0.118**

Note: The highest frequency of the data is in bold.

**Table 3:** Confirmatory factor analysis.

Model	X^2^	gl	X^2^/gl	CFI	TLI	SRMR	RMSEA (I.C. 95%)
Obtained (14 items)	398	77	5.16	0.96	0.95	0.038	0.07 (0.06–0.07)
Obtained (12 items)	252	54	4.66	0.97	0.97	0.023	0.06 (0.05–0.07)
Desired	---	---	<3.0	>0.90	>0.90	Close to 0	<0.10

**Table 4:** Configural invariance model of the scale.

	X^2^ (df)	ΔX^2^ (*p* > 0.05)	CMIN/df	CFI (>0.90)	ΔCFI (<0.010)	RMSEA (<0.100)	ΔRMSEA (<0.015)	Invariance (Trends)
Across gender								
Multigroup analysis								
Configural model	6.515 (11)	**0.837**	0.592	0.971	---	0.046	---	Invariant
Metric model	21.78 (23)	**0.533**	0.946	0.972	**0.001**	0.043	**0.003**	Invariant
Scalar model	21.79 (24)	**0.592**	0.908	0.971	**0.001**	0.047	**0.004**	Invariant
Strict model	40.938 (36)	**0.263**	1.138	0.970	**0.000**	0.045	**0.002**	Invariant
Across origin								
Multigroup analysis								
Configural model	8.53 (11)	**0.665**	0.775	0.970	---	0.047	---	Invariant
Metric model	61.71 (23)	**0.00**	2.68	0.971	**0.001**	0.044	**0.003**	Invariant
Scalar model	62.04 (24)	**0.00**	2.58	0.965	**0.006**	0.047	**0.003**	Invariant
Strict model	184.625 (36)	**0.00**	5.13	0.951	**0.014**	0.052	**0.005**	Invariant

Note: The highest frequency of the data is in bold.

## Data Availability

The data presented in this study are available on request from the corresponding author as they comprise information that compromises the privacy of research participants.

## Abbreviations

The following abbreviations are used in this manuscript:

ABC-CNS	ABC Connection with Nature Scale
CFA	Confirmatory Factor Analysis
CFI	Comparative Fit Index
CNI-PPC	Connection with Nature for Parents of Preschool Children
CNS	Connection with Nature Scale
EFA	Exploratory Factor Analysis
INS	Nature in Self Scale
NR	Nature Relatedness
NR-6	Nature Relatedness Scale
TLI	Tucker–Lewis’ Index

## Appendix A

Escala de Conectividad con la Naturaleza

Diseñada por Mayer y Frantz (2004) [12] y adaptada para contextos de habla hispana por Olivos, Aragonés y Amérigo (2011) [38]

Estimado participante,

A continuación, encontrará una serie de afirmaciones. Marque con una X, la valoración que usted considere más relevante para cada una de las afirmaciones. Por favor responda con sinceridad.

1. A menudo me siento en unión con el mundo natural que me rodea.
2. Pienso en el mundo natural como en la comunidad a la que pertenezco.
3. Reconozco y valoro la inteligencia de otros seres vivos.
4. Frecuentemente me siento desconectado de la naturaleza.
5. Cuando pienso en mi vida me imagino a mí mismo formando parte de un proceso cíclico más amplio de la vida.
6. A menudo me siento emparentado con los animales y plantas.
7. Siento como si perteneciera a la Tierra de la misma forma que ella me pertenece a mí.
8. Tengo una intensa comprensión de cómo mis actos afectan al mundo natural.
9. Frecuentemente me siento parte de la trama de la vida.
10. Siento que todos los habitantes de la Tierra, humanos y no humanos comparten una “fuerza vital” común.
11. De igual forma que el árbol forma parte del bosque, yo me siento incrustado dentro del mundo natural más amplio.
12. Cuando pienso en mi lugar en la Tierra, me considero a mí mismo como miembro de la cúspide jerárquica que existe en la naturaleza.
13. A menudo siento que sólo soy una pequeña parte del mundo natural que me rodea, y que no soy más importante que la hierba del suelo o los pájaros de los árboles.
14. Mi bienestar personal es independiente del bienestar del mundo natural.
